# A Retrocaecal Appendix Presenting With Recurrent Right Upper Quadrant Pain (RUQ): A Rare Presentation of a Rather Common Surgical Pathology

**DOI:** 10.7759/cureus.93826

**Published:** 2025-10-04

**Authors:** Ailsa Ferrie, Mohammed Barghash, Moustafa Mansour

**Affiliations:** 1 General Surgery, North Manchester General Hospital, Manchester, GBR

**Keywords:** appendicitis, chronic, computed tomography, laparoscopic, subhepatic

## Abstract

Acute appendicitis remains one of the most common causes of acute surgical abdomen. Variations in the anatomical position of the appendix can result in atypical clinical presentations with a variety of differential diagnoses and different diagnostic pathways being followed. This can ultimately lead to diagnostic delays with associated risks of increased complications. Subhepatic appendicitis is an exceptionally rare anatomical variant of acute appendicitis. We present the case of a 31-year-old female who reported symptoms of appendicitis but had a prior history of recurrent right upper quadrant (RUQ) pain in the absence of biliary pathology. Preoperative computed tomography (CT) of this lady revealed a long retrocecal appendix with its inflamed distal tip extending into the subhepatic space. CT imaging proved critical in identifying the cause of pain and the unusual anatomical position, thereby avoiding a potential missed diagnosis on ultrasound. Although rare, subhepatic appendicitis should be considered in patients presenting with right upper quadrant or atypical abdominal pains, especially when more common differentials, such as biliary disease, have been excluded. The patient underwent an uneventful laparoscopic appendectomy using a standard three-port technique without modification. We conclude that a high index of suspicion for variant positions of the appendix is required. Conventional laparoscopic port placement remains effective even in anatomical anomalies. Familiarity and experience with this approach may optimise surgical outcomes while minimising risks associated with altered techniques.

## Introduction

The appendix and its propensity to become acutely inflamed remains the most common cause of the acute surgical abdomen [1]. Appendectomy is a common surgical procedure; 50,000 are performed each year in the UK, despite conservative management becoming more popular [2]. Appendicitis is considered to be typical if there is a history of central colicky abdominal pain followed by sharp, well-localised pain in the right iliac fossa [3]. There is often also nausea, vomiting, and anorexia [3]. Atypical presentations include those occurring at the extremes of life, in pregnant women, and in association with various co-morbidities [3].

Of particular clinical concern are differences in presentation according to where the tip of the appendix lies, especially if in a rare location (e.g., lateral pouch, mesocolic, left-sided [4], intraherniary [5], subhepatic [3]). Atypical presentations can lead to a varied list of differential diagnoses, resulting in diagnostic delays and increased risk of complications [6]. Diagnostic imaging is helpful, particularly computed tomography (CT), which is widely used to confirm the diagnosis and to provide exact anatomical localisation [7]. 

In this article, we present a case of appendicitis where the appendix turned out to be a subhepatic variant on pre-operative imaging. The patient’s history of recurrent right upper quadrant pain further contributed to the diagnostic complexity.

Subsequent successful appendectomy with no modification to port placement was achieved with good surgical outcomes. As in our case, diagnostic laparoscopy offered additional clinical utility by permitting direct visualisation of intra-abdominal and gynaecological structures.

## Case presentation

A 31-year-old female presented to the Emergency Department with a 12-hour history of diffuse abdominal pain that was associated with nausea and multiple episodes of vomiting. She reported intermittent fever and denied any lower gastrointestinal and urinary symptoms. Her past medical history included asthma and gastro-oesophageal reflux disease. She had no previous surgical history and did not report any drug allergies. She was a non-smoker and reported occasional alcohol consumption.

She reported having had three prior episodes of colicky right upper quadrant (RUQ) pain and vomiting beginning 24 months prior to her last presentation. On each occasion, the pain was severe for approximately 6 to 8 hours before resolving spontaneously, leaving a residual ache in the right upper quadrant that persisted for several days. On one occasion, she had been seen in hospital and had had basic blood tests and an ultrasound (USS) of the abdomen, all of which were normal. Gallstone disease had been suspected but not confirmed.

Observations at presentation were normal and remained so. Her abdomen was soft but locally tender, with guarding and rebound tenderness in the right iliac fossa. She was also tender in the umbilical, right lumbar, and right upper quadrant regions. Rovsing and Psoas's signs were documented as positive [8].

**Figure 1:**
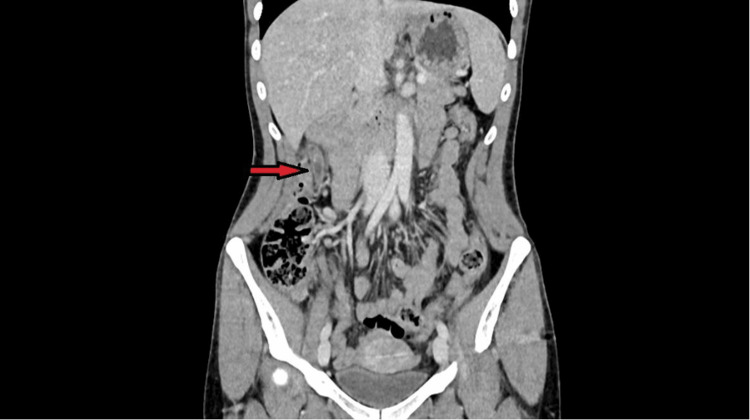
Coronal CT image showing the tip of the appendix located in the subhepatic region.

**Figure 2:**
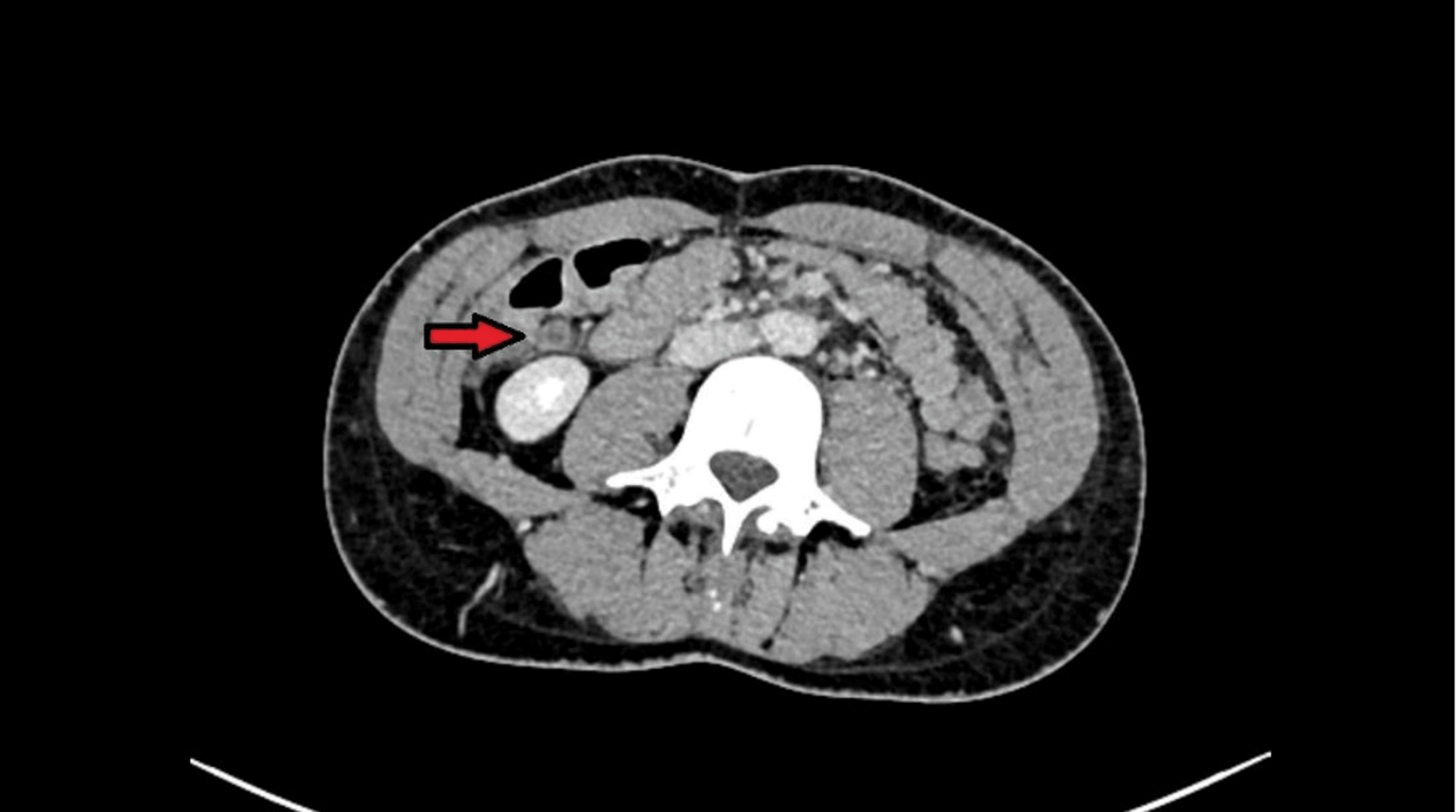
Axial abdominal CT scan image showing distended appendix with an enhancing wall and mild peri-appendiceal fat stranding.

All haematological and biochemical tests, including a urinary beta Human Chorionic Gonadotropin (HCG), were normal, but urinalysis was positive for blood. Acute appendicitis was considered in the differential diagnosis, with renal colic also a possibility. Therefore, a CT abdomen pelvis (CTAP) was requested. It showed a very long appendix, the distal 4 cm of which was mildly distended and inflamed, with enhancement of the wall and mild peri-appendiceal fat stranding (Figures 1, 2). There was also a small appendicolith proximal to this segment. These features were taken as confirmation of the diagnosis of acute appendicitis.

A laparoscopic appendectomy was performed under general anaesthesia using the classic three-port technique: 10 mm infra-umbilicus, 5 mm supra-pubic, and 5 mm left iliac fossa under direct vision.

Intra-operatively, there was a very long retrocaecal appendix running along the right paracolic gutter up to the hepatic flexure, with the tip in the subhepatic space. This was retrogradely dissected off the caecal pole, and adhesions were dissected along the ascending colon using blunt and sharp dissection.

The post-operative period was uneventful, and she was discharged home the following day on oral antibiotics. As of July 2025, she has had no further episodes of either right upper or lower quadrant pain.

## Discussion

Worldwide, in only 0.08% of cases is an inflamed appendix located subhepatically [9]. Surgeons should be aware of the variable positions of the appendix, as inflammation may present with atypical symptoms and signs. Its atypical presentation may contribute to diagnostic uncertainty, potentially resulting in delayed recognition and subsequent complications such as sepsis, abscess formation, and/or bowel perforation [10]. In light of this, knowledge of where the appendix lies is crucial to understanding, as it determines the clinical symptomatology, the diagnostic investigations to undertake, the surgical approach, and the likely potential complications and associated technical challenges. 

A subhepatic location for the appendix can be a consequence of congenital malformation [11] or, as in our case, an anatomical variant of an unusually long and mobile appendix, with its tip coming to lie beneath the liver. Congenital malformations include gut malrotation arising during intrauterine development and/or non-descent of the cecum into the typical right iliac fossa location [11]. This can be distinguished from cases like ours, in which the caecum is found in its typical location. There is no exact epidemiological evidence on what proportion of each cause contributes to the presentation due to limited literature information. Differentiation of the underlying aetiology using CT may be clinically valuable, as congenital causes are associated with a higher incidence of concurrent anatomical anomalies and other pathologies [12]. This distinction may influence surgical planning, not solely based on the severity of appendicitis, but also due to the need to address coexisting abnormalities.

It is important to be aware that an atypically located appendix can give rise to either typical and/or atypical signs and symptoms of appendicitis. Published cases of subhepatic appendicitis emphasise the importance of right upper quadrant pain and tenderness, which is unsurprising, given the location of the liver [10]. One possible explanation for this is the locality of the inflammatory reaction that is related to the inflamed tip. If the inflammatory process was confined to the proximal appendix, given the normal location of the base of the appendix in our patient, right iliac fossa pain and tenderness could be readily understood. If only the tip of the appendix were inflamed, the pain and tenderness would be expected to localise in the right upper quadrant. If the whole length of the appendix was inflamed, the pain might be expected to be more diffuse, involving the right iliac fossa and right hypochondrium as well as the right lumbar region.

Our patient had experienced recurrent episodes of right upper quadrant (RUQ) pain over the preceding two years. She has had no such attacks since her appendicectomy. The finding of a subhepatic appendix suggests that these episodes were a manifestation of a chronically inflamed subhepatic appendix. The absence of biliary pathology on both CTAP and USS imaging, the lack of biliary findings at surgery, and the absence of any further attacks of RUQ postoperatively confirm that her symptoms were highly likely related to her retrocaecal appendix. 

Chronic appendicitis is a recognised, albeit uncommon, cause of recurrent right iliac fossa pain, with recent estimates suggesting an incidence of approximately 1% [13]. A symptom duration exceeding seven days, whether recurrent or persistent, has been proposed as the criterion between acute and chronic appendicitis [14].

Isolated case reports, including our own, have described a chronic presentation of subhepatic appendicitis characterised by recurrent right upper quadrant pain. Notably, two of these cases involved patients under the age of 30, which aligns with the demographic of our case [15,16]. This may have contributed to a reduced likelihood of a comprehensive investigation. Despite presenting with RUQ pain, these individuals did not undergo imaging during previous episodes, potentially reflecting a lower index of clinical suspicion for biliary pathology given their age and a lack of consideration for a chronic appendicitis course. This is particularly relevant given the established propensity for subhepatic appendicitis to be misdiagnosed as either acute or chronic cholecystitis [15].

One case involved a middle-aged patient who had undergone a cholecystectomy one month prior to diagnosis yet continued to experience symptoms postoperatively [11]. This persistence ultimately led to the identification of chronic subhepatic appendicitis.

Our case highlights the diagnostic challenges posed by subhepatic appendicitis, particularly when USS yields negative findings. In contrast to the three previously reported cases, where USS successfully identified subhepatic appendicitis with a chronic course, our patient’s USS was negative. This discrepancy may be operator-dependent or could be attributed to the extent of inflammation: while the prior cases demonstrated widespread inflammatory involvement beyond the distal tip of the appendix, our case involved minimal or early-stage inflammation, limiting the sensitivity of USS.

Ultrasound remains the first-line imaging modality for suspected appendicitis due to its cost-effectiveness and lack of radiation [3]. However, in cases with atypical presentations or anatomical variations, ultrasound may delay definitive diagnosis and treatment, paradoxically increasing the risk of harm [10].

In our case, the clinical presentation mimicked renal or biliary colic according to the clerking doctors, prompting further imaging due to the presence of microscopic haematuria and normal inflammatory markers. CT has been proposed as a more reliable modality in such contexts, particularly when USS is inconclusive [17]. Therefore, this case underscores the importance of considering CT in all ultrasound-negative but otherwise typical presentations of appendicitis. Furthermore, atypically located appendices should remain within the differential diagnosis when clinical suspicion persists despite negative initial imaging.

Some centres dealing with similar cases of subhepatic appendicitis have adopted laparoscopy as an investigative modality, discovering the aberrant anatomical location whilst operating. The benefit of the pre-operative diagnosis of an anatomically variant appendix in our case allowed for appropriate pre-operative planning and ensured that the correct skill level of the surgeon was available. Surgical exploration of an anatomically rare appendix could result in longer operative time and/or a lack of appropriate experience available at the time of the operation.

In other case reports of subhepatic appendicitis, there have been calls to adopt a modified port approach or, if complications are known, such as a gangrenous or perforated appendix, an open technique [9,18].

The conventional laparoscopic appendectomy port placement consists of a 10 mm umbilical port with two 5 mm ports in the suprapubic region and left lower quadrant [19]. Modified techniques include moving one 5 mm port to the right lower quadrant or insertion of an additional 5 mm port. The decision in our case to adopt a standard port placement was successful and resulted in no post-operative complications.

A modified approach, of course, can be justified, but a surgeon should consider the benefits and potential implications of doing so. An extra port placement carries the additional risk of scarring (especially given an extended incision would be likely with a subhepatic location), hernia, or intra-abdominal injury [20]. If a surgeon is well-experienced with the conventional port placement, then you can extrapolate that familiarity is beneficial to surgical outcomes.

We propose that laparoscopic findings should dictate a surgical approach; hence, in a stable, non-complicated patient, standard port placement can be used with successful outcomes [14]. The benefit of laparoscopy is its versatility to be both a diagnostic and therapeutic tool. When required, modification of the technique and/or port placement can be adopted based on intraoperative findings and imaging.

## Conclusions

In this report, we presented a case of a young female who was found to have a long subhepatic appendicitis causing recurrent right upper quadrant pain. This case highlights the critical role of CT imaging to promptly aid diagnostic dilemmas and identify anatomically rare appendix locations. Clinicians should consider the possibility of chronic appendicitis with recurrent abdominal pain, especially when the top differentials have been excluded. The preoperative identification of an aberrantly located appendix facilitated surgical planning without dictating a specific operative approach. Laparoscopic appendectomy was successfully performed without necessitating modifications to port placement, underscoring the utility of laparoscopy as both a diagnostic and therapeutic tool. Applying extra ports always remains a viable option if the standard ports do not provide adequate access and/or exposure of the operative field.
